# Supplementary material for: Prescription Opioid Distribution after the Legalization of Recreational Marijuana in Colorado
Source: Int J Environ Res Public Health. 2020 May 7;17(9):3251. doi: 10.3390/ijerph17093251 (PMC7246665; doi:10.3390/ijerph17093251)

Supplemental Figure 1. Heat maps of prescription opioids by morphine mg equivalents (MME) as reported by the Drug Enforcement Administration's Automated Reports and Consolidated Ordering System by three-digit zip code.

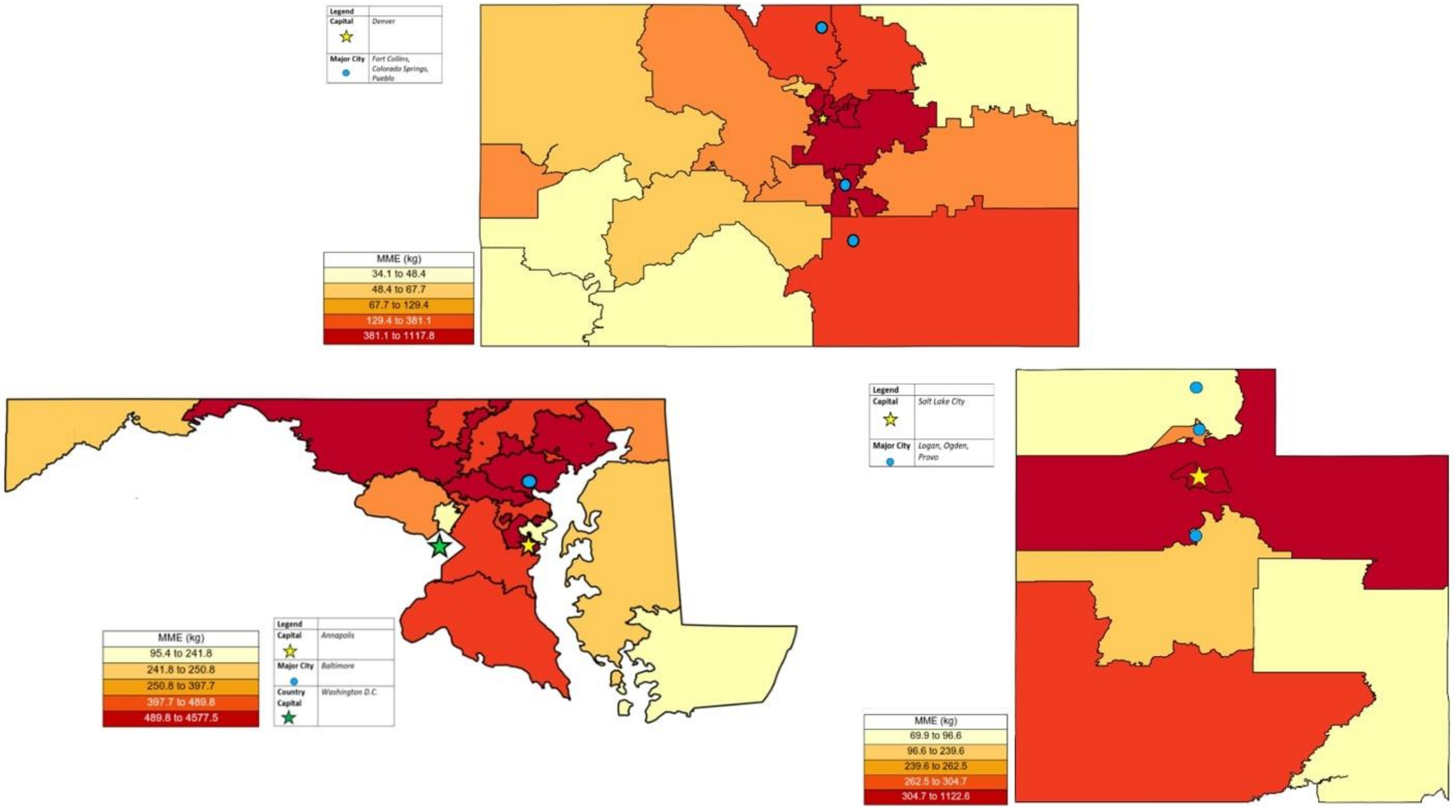

Supplement: Supplementary file 1 [file ijerph-17-03251-s001.pdf]
